# Ultra-processed food intake in toddlerhood and mid-childhood in the UK: cross sectional and longitudinal perspectives

**DOI:** 10.1007/s00394-024-03496-7

**Published:** 2024-10-04

**Authors:** Rana E. Conway, Gabriella N. Heuchan, Lisa Heggie, Fernanda Rauber, Natalie Lowry, Hannah Hallen, Clare H. Llewellyn

**Affiliations:** 1 Research Department of Behavioral Science and Health, University College London, London, UK; 2 Centre for Epidemiological Research in Nutrition and Health, University of São Paulo, São Paulo, Brazil; 3 Department of Preventive Medicine, School of Medicine, University of São Paulo, São Paulo, Brazil

**Keywords:** Ultra-processed foods, Diet quality, Toddlers, Children, UK

## Abstract

**Purpose:**

(i) Characterize ultra-processed food (UPF) intakes in toddlerhood and mid-childhood, including identifying principal UPF sub-groups and associations with nutrient profile; (ii) explore stability and change in UPF intake between toddlerhood and mid-childhood.

**Methods:**

Data were from children in the UK Gemini twin cohort at 21 months (*n* = 2,591) and 7 years (*n* = 592) of age. UPF intakes were estimated using diet diaries and Nova classification. Complex samples general linear or logistic regression models were used to explore associations between UPF intake, UPF sub-groups and nutrients, and changes in intake over time.

**Results:**

The contribution of UPF to total energy was 46.9% (± 14.7) at 21 months and 59.4% (± 12.5) at 7 years. Principal UPF sub-groups were yogurts, higher-fiber breakfast cereals, and wholegrain breads in toddlerhood, and puddings and sweet cereal products and white breads in mid-childhood. At both ages, mean free sugar and sodium intakes exceeded recommended maximums and higher UPF consumption was associated with consuming more of each nutrient (*P* < 0.001). UPF intake was negatively associated with fat, saturated fat and protein intake in toddlerhood, and fiber intake in mid-childhood (*P* < 0.001). Being in the highest UPF intake quintile in toddlerhood was predictive of being in the highest quintile in mid-childhood (OR 9.40, 95%CI 3.94–22.46).

**Conclusions:**

UPF accounted for nearly half of toddlers’ energy, increasing to 59% in mid-childhood. Higher UPF consumers had higher intakes of free sugar and sodium. UPF intake in toddlerhood was predictive of mid-childhood intake. Effective policies are needed to reduce UPF intakes in the early years.

**Supplementary Information:**

The online version contains supplementary material available at 10.1007/s00394-024-03496-7.

## Introduction

Encouraging toddlers and children to consume a varied and nutritious diet helps protect health in the early years and sets firm foundations for future health and wellbeing. National survey data indicates children in the United Kingdom (UK) fall short of dietary guidelines for many key nutrients. Most children aged 1.5-3 years (85%) and 4–10 years (98%) exceed the recommended maximum intake of 5% energy from free sugar [1, 2]. In addition, children in the UK consume less fiber and more saturated fat than recommended [2]. When children in England start school (aged 4–5 years), 22% have already developed overweight or obesity; this increases to 38% by the time children leave primary education (aged 10–11 years) [3].

Food preferences and eating behaviors are moderately heritable but are also notably shaped by early food experiences [4]. For example, repeated exposure to particular vegetables in infancy and toddlerhood has consistently been shown to increase the acceptability of the exposed vegetable and of vegetables in general [4]. This presents opportunities for encouraging healthy dietary habits. However, one study showed that trying a greater number of noncore foods (i.e., foods that are micronutrient-poor but high in fat, sugar or salt) at age 14 months was positively associated with preference and intake of these foods at age 3.7 years [5]. Given the well-documented relationship between early food-related behaviors, dietary patterns and medium- to long-term health, establishing healthy preferences and dietary patterns in early life should be a public health priority [6].

There is increasing interest in the role that industrial food processing plays in the diets of both children and adults. Ultra-processed foods (UPFs), a category outlined in the Nova classification, are defined as industrial formulations created through the deconstruction of whole foods into food-derived substances (e.g., fats, sugars, starches, isolated proteins), which are then modified and recombined with additives such as colourants, flavourings, and emulsifiers to produce final products [7]. Associations between higher UPF intake and poorer health outcomes, including obesity, have consistently been demonstrated in observational studies with both adult and child populations [8, 9]. One potential mechanism underpinning this association is the unfavourable nutrient profile of diets comprising greater amounts of UPF [10]. In a meta-analysis of observational data including adults and children from 13 high- and middle-income countries, increased UPF intake was associated with higher consumption of energy, fat, saturated fat and free sugar, and lower intake of fiber, protein, potassium, vitamin C and many other micronutrients [11]. The meta-analysis also found that as dietary share of UPF increased, intake of health-protective foods reduced, including fruit, vegetables, beans and legumes [11]. Another meta-analysis of 8 countries’ national survey data by Neri et al. found that, across 3 childhood age groups (pre-school, primary school and high school), higher intake of UPF was associated with the type of nutrient profile commonly associated with obesity, i.e., higher energy density and free sugar and lower fiber [12]. A notable unexplained exception was that no association was found between UPF and fiber in UK pre-school children (2–5 years), which may point to the differing eating patterns of the youngest pre-schoolers compared with older children, as they are still transitioning from predominantly milk-based diets [12].

There are limited data available that specifically examine UPF intake in toddlerhood. A study of 360 toddlers (aged 12–24 months) in Brazil found that processed foods and UPFs together represented 34.8% of total energy (%E) [13]. The UK National Diet and Nutrition Survey (NDNS) sample included in Neri et al.’s meta-analysis comprised few 2 year olds (*n* < 300) and did not extend to 1 year olds [12]. There is also limited information regarding tracking of UPF intake throughout childhood. Moderate longitudinal stability in UPF consumption was observed between the ages of 4–7 years in the Portuguese Generation XXI study (*n* = 1175, Pearson’s correlation coefficient = 0.34) [14]. Stability in UPF intake during the more pronounced developmental period from toddlerhood to mid-childhood is less clear, but is important to examine given enduring food preferences can be formed during the first two to three years of life [6].

Infants and young children are recognized as a more vulnerable group nutritionally and, as such, commercial food products marketed for those up to 36 months of age are more tightly regulated than those for older consumers [15]. UPFs given to toddlers may differ from those chosen by older consumers for consumption themselves. In fact, in a recent qualitative study, UK parents reported using commercial infant foods rather than home-prepared foods, as they felt more confident these would contain the appropriate balance of nutrients [16]. However, a survey of 3,427 commercial infant foods sold in Europe found 29% were UPFs and, compared to less processed products, these were typically more energy dense and higher in fat, saturated fat, sugar and sodium [17]. Prolonged feeding of commercial milk formula (CMF) - classified as a UPF under Nova - is not recommended beyond 12 months of age and is associated with parental concern over inadequate nutrient intake [1, 18].

This study aimed to (i) characterize UPF intakes in toddlerhood and mid-childhood, including identifying principal UPF sub-groups and association with nutrient profile, and (ii) explore stability and change in UPF intake between toddlerhood and mid-childhood.

## Methods

### Study design and population

Children were from Gemini, a UK population-based birth cohort of families with twins. In 2007–2008, the Office for National Statistics contacted all families in England and Wales who had live twin births between March and December 2007 (*n* = 6,754 families) and asked for their permission to be contacted by the research team at University College London (UCL). Approximately half of the sample consented to be contacted (*n* = 3,435 families), with 2,402 families (*n* = 4,804 children) providing written informed consent and baseline questionnaires when children were on average 8.2 ± 2.2 months of age (36% of all live twin births within this period in England and Wales) [19].

In the baseline questionnaire, parents reported the sex, date of birth, gestational age, and birth weight of children. Maternal ethnicity was recorded and dichotomized into white and non-white. Parents provided information about seven indicators of socio-economic status (SES) which were used to create a composite SES score [20]. The study was approved by the UCL Committee for the Ethics of non-National Health Service Human Research.

### UPF consumption

Diet diaries were mailed to all families between November 2008 and August 2009 (4,804 children) [21]. Between April and December 2014, a second diet diary was mailed to families who were still engaged with the Gemini study (3,690 children). The dietary data collection and coding methodology used in toddlerhood have been reported previously; the same approaches were used in mid-childhood [21]. In brief, parents were asked to prospectively record all food, drink and supplements consumed by both children for two weekdays and one weekend day. Families were provided with a paper diary and instructions, along with age-appropriate pictorial food and portion guides. All diaries collected for toddlers and diaries collected for 200 children were coded at the University of Cambridge. The remaining diet diaries were coded at UCL. An age-specific coding system was used with DINO (Diet In, Nutrients Out) dietary assessment software [22]. Diet diaries were returned for 2,714 toddlers and 615 children. 122 of the toddler diaries and 23 of the mid-childhood diaries were excluded as they were completed for less than two full days. In total, 90.1% of toddler diaries and 94.9% of mid-childhood diaries were 3-day records. Diaries were accepted irrespective of whether families reported a weekend day.

Nova classification was used to determine UPF intake. Food and drink items in the DINO database consumed by Gemini children were classified into one of four Nova groups: minimally/unprocessed food (group 1), culinary ingredients (group 2), processed food (group 3) or UPF (group 4) [7, 23, 24]. Where necessary, ingredient lists for commercially prepared foods were checked to determine the appropriate classification. Four researchers (HH, LH, NL and RC) independently classified all foods and where differences were found, these were discussed. Items that the four researchers felt uncertain about were discussed with FR who is experienced with using Nova classification. A list of rules was compiled to ensure consistent classification of similar items (supplementary material 1). Following each group discussion, the four researchers reviewed their individual classification of items and reclassified items until consensus was reached for all items. A small number of additional foods were later classified by consensus between GH and RC using the same principles, as additional diet diaries were coded after the initial process was completed.

The Nova group classification for each food was integrated with the DINO food composition database. UPF sub-groups reflect only the items classified as UPF within a food group, for example the UPF sub-group ‘Infant foods and drinks’ includes commercially produced products with added flavouring or emulsifier but excludes those containing only pureed fruit. Consumption of UPF was computed as %E. Dietary supplements, such as vitamins, minerals and fish oils were excluded from this analysis.

### Statistical analysis

Sociodemographic characteristics of the sample completing diet records at 21 months were compared with the baseline Gemini sample using Pearson’s chi-squared tests for categorical variables and t-tests for continuous variables. These analyses were repeated for the sample completing diet records at 7 years.

UPF intake (%E) was evaluated for the complete sample and children were also categorized into quintiles of UPF intake (%E). To identify UPF sub-groups making the largest contribution to energy intake at each time point, those contributing a mean of more than 1%E at either time point were identified. Complex samples general linear models (CSGLM) were performed to account for clustering of twin data within families. Separate models tested associations between %E from UPF (as a continuous independent variable) and %E from UPF sub-groups, energy, fat, saturated fat, carbohydrate, free sugar, protein, fibre and sodium (as continuous dependent variables). The nutrients selected are of particular concern in the context of UK children’s diets and age-appropriate UK guidelines are presented for reference [1, 25–28]. As some toddlers consumed large amounts of cows’ milk, it was included in the analysis as a separate variable, even though it is not a UPF. As CMF contributed a substantial proportion of energy to some toddlers’ diets, analyses were repeated excluding individuals consuming any CMF. CSGLM were also used to analyse associations between UPF intake in toddlerhood and mid-childhood, with both as continuous variables. As this was exploratory analysis, models did not include covariates. Associations between quintile of UPF intake in toddlerhood and being in the highest UPF quintile in mid-childhood were determined using complex samples logistic regression. Statistical analyses were performed using SPSS 27.

## Results

**Fig. 1:**
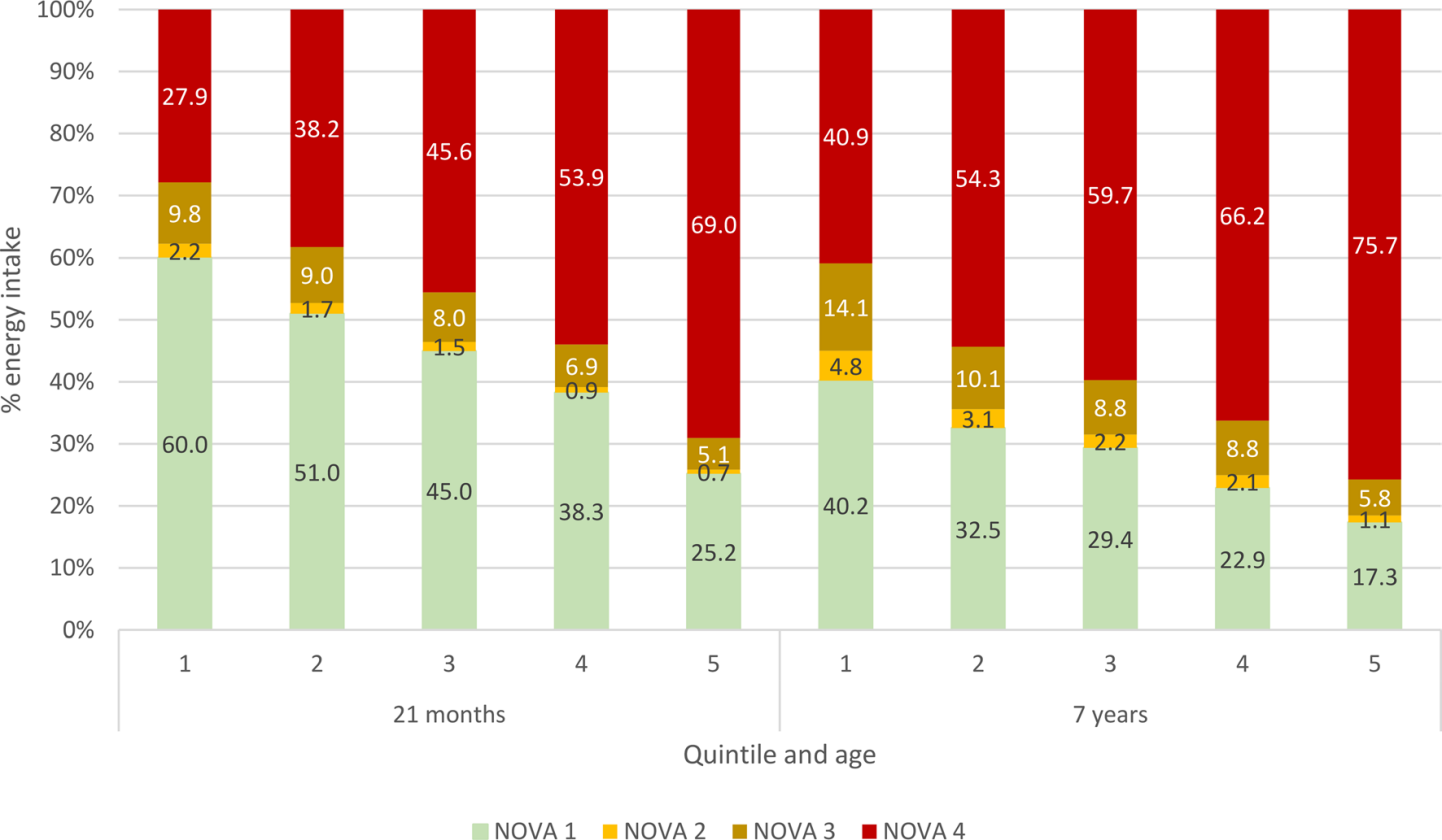
Contribution of Nova food groups to total energy at 21 months (*n* = 2591) and 7 years (*n* = 592)

Diet records were completed for 2,592 toddlers. Data for one toddler, who consumed an energy-dense supplement, were excluded. This analysis therefore includes 2,591 toddlers and 592 children, including 570 individuals with data at both time points. The samples included similar proportions of boys and girls and most children were of white ethnicity (Table 1). Compared to the baseline Gemini sample, both samples had a higher proportion of families with white ethnic backgrounds and mean SES scores were higher. Mean energy intake was 1,033 kcal/day (± 187) in toddlerhood and 1,500 kcal/day (± 259) in mid-childhood. UPFs contributed 46.9%E (± 14.7) at 21 months and 59.4%E (± 12.5) at 7 years. Figure 1 shows the proportion of energy derived from each Nova group according to quintile of UPF intake. Considerable variation in intake of Nova 1 (%E) was seen across the UPF quintiles. The distribution of UPF intake was wider in toddlerhood than mid-childhood, with UPF contributing 41.2% more energy in quintile 5 than quintile 1 in toddlerhood (69.0%E and 27.9%E, respectively), compared to a difference of 34.8% between quintiles 5 and 1 in mid-childhood (75.7%E and 40.9%E, respectively).

**Table 1:** Sociodemographic characteristics in toddlerhood and mid-childhood compared to baseline

Characteristic	Baseline^a^ (*n* = 4,804)	21m^b^ (*n* = 2,591)	*p*-value	7y^c^ (*n* = 592)	*p*-value
Sex, n (%)					
Boys	2,386 (49.7)	1,277 (49.3)		292 (49.3)	
Girls	2,418 (50.3)	1,314 (50.7)	0.755^d^	300 (50.7)	0.875^d^
Ethnicity, n (%)					
White	4,462 (92.9)	2,457 (94.8)		572 (96.6)	
Non-white	338 (7.0)	134 (5.2)	0.002^d^	20 (3.4)	< 0.001^a^
Not known	4 (0.1)	-		-	
Socio-economic status, mean (SD)^e^	4.3 (1.4)	4.6 (1.3)	< 0.001^f^	4.9 (1.1)	< 0.001^f^
Age at baseline (m), mean (SD)		7.9 (2.0)	< 0.001^f^	7.7 (1.9)	< 0.001^f^
Age at diary (m), mean (SD)	-	20.9 (1.1)		85.3 (2.6)	
Gestational age (wk), mean (SD)	36.2 (2.5)	36.2 (2.5)	0.787^f^	36.4 (2.4)	0.164^f^
Birth weight (kg), mean (SD)	2.5 (0.5)	2.5 (0.5)	0.826 ^f^	2.5 (0.5)	0.919^f^
Birth weight SDS (kg), mean (SD)	-0.6 (0.9)	-0.5 (0.9)	0.744 ^f^	-0.6 (0.9)	0.356^f^

kg, kilograms; m, months; SDS, standard deviation score; wk, weeks

^a^At baseline, some children were missing data: maternal ethnicity, *n* = 4; SES, *n* = 278; gestational age, *n* = 20; weight at birth (kg), *n* = 165; and birth weight SDS (kg), *n* = 181

^b^At 21-months, some children were missing data: SES, *n* = 126; gestational age, *n* = 4; weight at birth (kg), *n* = 57; and birth weight SDS (kg), *n* = 61. ^c^At 7 years, some children were missing data: SES, *n* = 28; gestational age, *n* = 2; weight at birth (kg), *n* = 15; and birth weight SDS (kg), *n* = 17

^d^ Pearson’s chi-squared test to compare categorical characteristics between baseline Gemini sample and dietary data samples

^e^ Composite Socio-economic Status score. Scores ranged from 1.30 to 6.96, with higher scores reflecting higher SES [20]

^f^Independent samples t-test of differences between baseline Gemini sample and dietary data samples

Sub-groups of UPF making the largest contribution to toddlers’ energy intake included flavoured yogurts, higher-fiber breakfast cereals and wholegrain breads (Table 2). In the smaller group of participants who completed diaries in mid-childhood, UPFs contributing most energy were puddings and sweet cereal products, and white breads, followed by wholegrain breads, confectionary and biscuits. UPF intake was positively associated with intake of most UPF sub-groups (*P* < 0.001). However, UPF infant foods and drinks was an exception in toddlerhood, and UPF higher-fiber breakfast cereals and wholegrain breads were notable exceptions at both time points – intake of these UPF sub-groups was not associated with total UPF intake. CMF intake varied considerably, providing 0.3%E for toddlers in the lowest quintile (Q1) for UPF, but 9.2%E in the highest quintile (Q5). Whole milk intake by direct contrast provided 29.1%E for toddlers in Q1, compared with 9.2%E for those in Q5.

**Table 2:** Contribution of UPF, UPF sub-groups and whole milk to total energy intake (mean ± SD) for complete sample and by quintile of UPF intake in toddlerhood (21 months) and mid-childhood (7 years)

	Age	Total	Quintile of % of energy from UPF				
		(21 m: *n* = 2,591 7y: *n* = 592)	Q1 (21 m: *n* = 518 7y: *n* = 118)	Q2 (21 m: *n* = 519 7y: *n* = 119)	Q3 (21 m: *n* = 518 7y: *n* = 118)	Q4 (21 m: *n* = 517 7y: *n* = 119)	Q5 (21 m: *n* = 519 7y: *n* = 118)
**UPF** (% energy)	21 m	46.9 ± 14.7	27.9 ± 5.6	38.2 ± 2.0	45.6 ± 2.1	53.9 ± 2.9	69.0 ± 7.4
	7y	59.4 ± 12.5	40.9 ± 7.9	54.3 ± 1.8	59.7 ± 1.6	66.2 ± 2.0	75.7 ± 4.7
**Sub-group of UPF** ^a^							
Higher fiber breakfast cereals	21 m	4.2 ± 3.9	3.4 ± 3.2	4.8 ± 4.1	4.6 ± 4.0	4.0 ± 3.8	4.1 ± 4.2
	7y	3.4 ± 4.0	2.6 ± 3.0	3.9 ± 4.2	4.0 ± 4.2	3.6 ± 4.5	2.9 ± 3.8
Lower fiber breakfast cereals	21m^b^	0.9 ± 1.9	0.6 ± 1.4	0.7 ± 1.5	0.9 ± 1.8	1.3 ± 2.3	1.2 ± 2.3
	7y^b^	2.1 ± 3.1	1.3 ± 2.1	2.0 ± 2.9	2.2 ± 3.6	2.5 ± 3.5	2.6 ± 3.2
Wholegrain breads	21 m	3.9 ± 4.3	3.6 ± 3.5	4.2 ± 3.0	4.0 ± 4.1	4.0 ± 4.6	4.0 ± 5.4
	7y	4.9 ± 5.1	5.4 ± 5.1	5.1 ± 5.1	4.8 ± 4.5	5.0 ± 5.0	4.1 ± 5.5
White breads	21m^b^	3.7 ± 4.4	2.2 ± 3.1	2.9 ± 3.5	3.7 ± 4.2	4.8 ± 4.9	5.0 ± 5.2
	7y	6.5 ± 5.6	5.2 ± 4.7	6.6 ± 5.9	6.6 ± 5.9	6.3 ± 5.2	7.6 ± 5.8
Flavoured yogurts	21m^b^	4.5 ± 3.7	3.2 ± 3.1	4.3 ± 3.2	4.6 ± 3.4	5.1 ± 3.6	5.2 ± 4.5
	7y	2.4 ± 2.8	1.5 ± 2.3	2.5 ± 2.8	2.6 ± 2.7	2.8 ± 2.8	2.6 ± 3.1
Commercial Milk Formula	21m^b^	2.8 ± 7.9	0.3 ± 2.0	0.5 ± 2.5	1.1 ± 4.6	2.8 ± 7.1	9.2 ± 13.2
	7y	-	-	-	-	-	-
Infant foods and drinks	21 m	1.9 ± 3.6	1.9 ± 3.2	1.9 ± 3.0	1.7 ± 3.3	1.8 ± 3.5	2.1 ± 4.8
	7y	0.1 ± 0.6	0.1 ± 0.3	0.04 ± 0.3	0.1 ± 0.6	0.1 ± 0.9	0.1 ± 0.5
Puddings and sweet cereal products	21m^b^	3.1 ± 4.3	1.5 ± 2.7	2.6 ± 3.4	3.7 ± 4.6	3.8 ± 4.6	3.7 ± 5.0
	7y^b^	6.6 ± 6.2	4.2 ± 4.7	5.3 ± 5.4	5.8 ± 5.7	7.9 ± 5.8	9.7 ± 7.6
Ice cream, dairy desserts and ice lollies	21m^b^	1.0 ± 2.2	0.5 ± 1.4	0.7 ± 1.6	1.1 ± 2.1	1.2 ± 2.3	1.6 ± 3.0
	7y^b^	3.0 ± 3.4	1.7 ± 2.7	3.1 ± 3.4	3.0 ± 3.3	3.8 ± 3.4	3.4 ± 3.7
Biscuits	21m^b^	2.8 ± 3.7	1.5 ± 2.3	2.1 ± 2.5	2.7 ± 3.2	3.2 ± 3.4	4.7 ± 5.3
	7y^b^	3.9 ± 4.0	2.2 ± 3.0	3.0 ± 3.8	4.0 ± 3.6	4.9 ± 4.1	5.3 ± 4.5
Confectionery	21m^b^	1.5 ± 2.7	0.5 ± 1.2	1.0 ± 2.2	1.5 ± 2.5	1.9 ± 2.7	2.4 ± 3.6
	7y^b^	4.0 ± 4.1	2.9 ± 3.3	3.3 ± 3.8	4.3 ± 4.5	4.5 ± 3.8	4.8 ± 4.7
Sweet spreads	21m^b^	0.5 ± 1.2	0.3 ± 0.7	0.4 ± 0.8	0.5 ± 1.2	0.6 ± 1.3	0.7 ± 1.8
	7y	1.1 ± 1.8	0.7 ± 1.1	0.9 ± 1.7	0.9 ± 1.3	1.4 ± 2.3	1.4 ± 2.3
Savoury snacks	21m^b^	2.1 ± 2.8	1.1 ± 1.7	1.4 ± 1.9	2.2 ± 2.8	2.6 ± 2.9	3.4 ± 3.7
	7y^b^	3.2 ± 3.3	2.1 ± 2.7	3.0 ± 2.8	2.8 ± 3.1	3.4 ± 3.5	4.5 ± 3.8
Processed meat	21m^b^	1.7 ± 2.7	0.9 ± 1.6	1.2 ± 1.9	1.8 ± 2.6	1.9 ± 2.8	2.5 ± 3.7
	7y^b^	3.2 ± 3.4	2.3 ± 2.8	2.6 ± 3.1	3.3 ± 3.4	3.6 ± 3.8	4.4 ± 3.7
Poultry products & dishes	21m^b^	0.7 ± 2.0	0.1 ± 0.8	0.3 ± 1.1	0.5 ± 1.5	1.0 ± 2.1	1.4 ± 3.2
	7y^b^	1.2 ± 2.7	0.3 ± 1.2	1.0 ± 2.2	1.2 ± 2.4	1.7 ± 3.8	1.7 ± 3.0
Fish & fish dishes	21m^b^	1.3 ± 2.2	0.8 ± 1.6	1.3 ± 2.1	1.2 ± 2.1	1.7 ± 2.3	1.8 ± 2.6
	7y	1.4 ± 2.4	0.9 ± 2.1	1.1 ± 2.0	1.4 ± 2.0	1.6 ± 2.2	2.2 ± 3.4
Potato products, e.g. fries, wedges, instant mash	21m^b^	1.2 ± 2.3	0.5 ± 1.4	0.6 ± 1.4	1.0 ± 2.1	1.4 ± 2.2	2.5 ± 3.5
	7y^b^	2.1 ± 2.9	0.7 ± 1.6	1.5 ± 2.4	1.9 ± 25	2.6 ± 3.1	3.8 ± 3.7
Savoury pastry dishes	21m^b^	0.7 ± 2.4	0.3 ± 1.3	0.6 ± 1.8	0.6 ± 1.7	0.7 ± 1.8	1.5 ± 4.1
	7y	1.3 ± 2.8	0.6 ± 1.5	1.3 ± 3.2	1.3 ± 2.7	1.3 ± 3.0	1.9 ± 3.1
Pizza	21m^b^	0.6 ± 2.3	0.3 ± 1.2	0.5 ± 1.9	0.5 ± 2.0	0.9 ± 2.9	1.0 ± 3.1
	7y^b^	1.8 ± 3.8	0.5 ± 2.0	0.9 ± 2.4	1.6 ± 3.8	2.1 ± 4.0	3.7 ± 5.3
Spreadable fats	21m^b^	1.9 ± 2.4	0.9 ± 1.4	1.5 ± 1.9	2.1 ± 2.4	2.6 ± 2.7	2.7 ± 3.0
	7y	1.6 ± 2.0	1.0 ± 1.6	1.5 ± 2.0	1.9 ± 2.1	1.7 ± 2.0	1.8 ± 2.0
**Whole milk**	21m^c^	21.3 ± 12.5	29.1 ± 12.2	26.3 ± 10.7	23.6 ± 9.9	18.5 ± 9.9	9.2 ± 8.6
	7y	2.5 ± 5.4	4.1 ± 7.2	2.7 ± 5.6	2.8 ± 5.9	1.4 ± 3.1	1.6 ± 3.9

m, months; Q, quintile; UPF, ultra-processed food; y, years

^a^UPF sub-groups contributing > 1% energy included, therefore sum of sub-groups does not yield the value of UPF contribution

^b^Positive linear association (Complex Samples General Linear Models) between % energy from UPF (continuous) and % energy from UPF sub-group (continuous), *P* < 0.001

^c^Negative linear association (Complex Samples General Linear Models) between % energy from UPF (continuous) and % energy from UPF sub-group (continuous), *P* < 0.001

At both time points, there was a positive linear association between UPF intake (continuous) and free sugar and sodium (*P* < 0.001) (Table 3). Toddlers consuming more UPF were found to consume less fat, saturated fat and protein (*P* < 0.001). Although UPF intake was not associated with fiber intake in toddlerhood, a negative association was found in mid-childhood (*P* < 0.001). When analyses for toddlerhood were repeated excluding individuals consuming CMF (supplementary Table 2), results were similar, but a negative linear association was observed between UPF intake and fiber (*P* < 0.001).

**Table 3:** Daily energy and nutrient intake (mean ± SD) according to quintile of UPF intake in toddlerhood (21 months) and mid-childhood (7 years)

	Age	UK Guideline	Total	Quintile of % of energy from UPF				
			(21 m: *n* = 2,591 7y: *n* = 592)	Q1 (21 m: *n* = 518 7y: *n* = 118)	Q2 (21 m: *n* = 519 7y: *n* = 119)	Q3 (21 m: *n* = 518 7y: *n* = 118)	Q4 (21 m: *n* = 517 7y: *n* = 119)	Q5 (21 m: *n* = 519 7y: *n* = 118)
Energy (kcals)	21 m	944^c^	1033 ± 187	1027 ± 180	1042 ± 173	1044 ± 170	1044 ± 208	1011 ± 199
	7y	1768^c^	1500 ± 259	1442 ± 247	1494 ± 256	1529 ± 280	1486 ± 238	1556 ± 261
Fat (%E)	21m^b^	^d^	36.2 ± 4.8	37.6 ± 5.0	36.4 ± 4.5	36.6 ± 4.5	35.5 ± 4.8	34.7 ± 4.7
	7y	≤ 33^d^	33.3 ± 5.0	34.1 ± 5.1	32.5 ± 5.1	33.1 ± 5.1	33.1 ± 4.5	34.0 ± 4.9
Saturated fat (%E)	21m^b^	^d^	15.7 ± 3.5	17.3 ± 3.5	16.3 ± 3.3	16.3 ± 3.1	15.0 ± 3.3	13.6 ± 3.2
	7y	≤ 10^d^	13.2 ± 2.9	13.6 ± 3.1	12.9 ± 3.4	13.4 ± 2.8	13.0 ± 2.4	13.2 ± 2.7
Carbohydrate (%E)	21m^a^	50^e^	48.4 ± 5.5	46.0 ± 5.7	47.3 ± 5.0	47.6 ± 4.7	49.4 ± 5.1	51.6 ± 5.2
	7y	50^e^	52.3 ± 5.1	50.9 ± 5.3	52.5 ± 4.8	52.3 ± 5.1	53.2 ± 4.6	52.8 ± 5.4
Free sugar (%E)	21m^a^	≤ 5^e^	9.3 ± 4.6	6.4 ± 3.5	7.8 ± 3.5	8.9 ± 3.6	10.3 ± 4.1	13.0 ± 5.3
	7y^a^	≤ 5^e^	14.1 ± 4.9	11.1 ± 4.3	13.5 ± 5.0	13.9 ± 4.1	15.6 ± 4.2	16.1 ± 5.0
Protein (g)	21m^b^	14.5^f^	40.0 ± 8.6	42.3 ± 8.3	42.4 ± 7.7	41.3 ± 8.0	39.3 ± 8.5	34.5 ± 8.1
	7y	28.3^f^	53.7 ± 10.9	54.3 ± 10.3	55.9 ± 11.1	56.0 ± 11.9	50.8 ± 10.2	51.3 ± 9.7
Fiber (g)	21 m	^g^	9.4 ± 3.0	9.5 ± 3.0	9.7 ± 3.1	9.0 ± 2.8	9.2 ± 3.0	9.3 ± 3.3
	7y^b^	20^g^	10.9 ± 2.8	12.6 ± 3.3	11.3 ± 2.8	10.9 ± 2.5	10.3 ± 2.1	9.4 ± 2.2
Sodium (mg)	21m^a^	500^h^	1112 ± 338	925 ± 262	1059 ± 263	1145 ± 306	1206 ± 359	1223 ± 388
	7y^a^	1200^h^	1778 ± 451	1607 ± 385	1699 ± 440	1838 ± 493	1760 ± 377	1988 ± 451

%E = percentage of total energy; m = months; UPF, ultra-processed food; y = years

^a^ Positive linear association (CSGLM) between % energy from UPF (continuous) and nutrient (continuous), *P* < 0.001

^b^ Negative linear association (CSGLM) between % energy from UPF (continuous) and nutrient (continuous), *P* < 0.001

^c^Mean of Estimated Average Requirement for Energy for boys and girls aged 1–3 years and 7–10 years respectively [25]

^d^Dietary Reference Values for fat and saturated fat as % Total Dietary Energy Intake do not apply before 2 years and apply in full from age 5 years [1]

^e^Population guideline from 1 year of age [1, 26]

^f^Reference Nutrient Intake for children aged 1–3 years and 7–10 years respectively [27]

^g^No fibre recommendation for children below 2 years of age, 15 g/day for children aged 2-5years and 20 g/day for children aged 5–11 years [26]

^h^Reference Nutrient Intake for children aged 1–3 years and 7–10 years respectively [28]

A substantial increase in UPF contribution to %E was seen between toddlerhood and mid-childhood (Table 4). Individuals completing diet records at both time points (*n* = 570) increased UPF intake on average by 15.7%E between toddlerhood and mid-childhood. Repeating this analysis for the 467 individuals who did not consume CMF in toddlerhood showed an increase in UPF intake of 17.6%E between the two ages. UPF intake in toddlerhood accounted for 19.4% of the variance in mid-childhood UPF intake for the complete sample and 21.8% of the variance when toddlers consuming CMF were excluded (*P* < 0.001). CMF consumers had a smaller, but significant, increase in UPF intake in mid-childhood (7.0%). Being in Q4 or Q5 as a toddler increased the likelihood of being in Q5 for UPF intake in mid-childhood, and the odds ratio for toddlers in Q5 progressing on to be in Q5 in mid-childhood increased from 9.40 to 21.51 when CMF consumers were excluded (Table 5).

**Table 4:** Association between UPF intake in toddlerhood and mid-childhood in the subsample of children with diet records at both ages

Sample		UPF intake at 21m^a^		UPF intake at 7y^a^		Increase between 21 m and 7y^a^				
	n	mean	(95% CI)	mean	(95% CI)	mean	(95% CI)	B (SE)	P	R^2^
Complete sample	570	43.6	42.0-45.2	59.3	57.9–60.7	15.7	14.5–16.9	0.388 (0.049)	< 0.001	0.194
Non-consumers of CMF	467	41.7	40.0-43.4	59.4	57.8–61.0	17.6	16.0-19.3	0.450 (0.52)	< 0.001	0.218
Consumers of CMF	103	52.1	47.9–56.0	59.1	56.0-62.2	7.0	3.0-10.9	0.335 (0.113)	0.004	0.194

B, unstandardised beta; CI, Confidence Interval; m, months; CMF, Commercial Milk Formula; UPF, ultra-processed food; m, months; y, years

^a^CSGLM with UPF intake (% energy) at 21 m (continuous independent variable) and UPF intake (% energy) at 7y (continuous dependent variable)

**Table 5:** Association between quintile of UPF intake (%E) in toddlerhood and being in the highest quintile (Q5) for UPF intake in mid-childhood in the subsample of children with diet records at both ages^a^

Quintile of UPF intake in toddlerhood	Model 1. Complete sample (consumers and non-consumers of CMF)^b^ (*n* = 114)			Model 2. Non-consumers of CMF (Consumers of CMF excluded)^b^ (*n* = 100)		
	Children in highest UPF quintile at 7 y n (%)	OR	95% CI	Children in highest UPF quintile at 7 y n (%)	OR	95% CI
1	15 (10.1)	1.0		15 (11.0)	1.0	
2	16 (13.0)	1.34	0.52–3.44	14 (13.0)	1.20	0.44–3.25
3	21 (17.4)	1.88	0.78–4.54	20 (19.6)	1.97	0.80–4.84
4	22 (22.0)	2.55	1.038–6.274	19 (24.7)	2.64	1.027-6.80
5	40 (51.3)	9.40	3.94–22.46	32 (72.1)	21.51	7.89–58.64

CI, Confidence Interval; CMF, commercial milk formula; %E, percentage of total energy; m = months; OR, Odds Ratio; UPF, ultra-processed food; y, years

^a^ Data are presented only for children in Q5 in mid-childhood

^b^Complex samples logistic regression, *P* < 0.001 for both models

## Discussion

This study estimated UPF intake in toddlerhood and mid-childhood and explored stability and change in intakes between the two time points. UPF accounted for nearly half of total energy in toddlerhood (47%E), and 59%E in mid-childhood. Higher UPF consumers had higher intakes of free sugar and sodium, and UPF intake in toddlerhood was found to be predictive of mid-childhood intake.

Gemini toddlers’ energy and nutrient intake has been reported previously and is broadly similar to that of the much smaller sample of children aged 18–36 months in the UK national survey [21]. Intakes in mid-childhood have not been presented previously. In the current study, the contribution of UPF to total energy intake in toddlerhood (47%) was less than that of 2-5-year-olds in the UK national survey (61%) [12]. This is expected as although national survey data was collected at a comparable time point, the sample was more diverse in terms of ethnicity and SES, as well as covering a wider age range which spanned both toddlerhood and early childhood [12]. Likewise, UPF intake by Gemini children at age 7 years (59%) was lower than that reported in national survey data for a representative sample of UK children aged 6–11 years (67%) [12]. Some, although not all, studies have found a positive association between socioeconomic disadvantage and UPF intake, and the overrepresentation of children from higher SES households in the Gemini cohort compared to the UK population, along with age differences, may partially explain the lower observed UPF intakes [29, 30]. The higher dietary share of UPF in mid-childhood compared to toddlerhood is in line with cross-sectional observations described by Neri et al. comparing national survey data between pre-school, primary school and high school aged children in the UK, United States and Australia [12]. While an increase in UPF intake of 15.7% was observed in our longitudinal analysis, a smaller increase of 2% was observed among Portuguese children when they were aged 7 years compared to 4 years [14, 31]. A linear association between UPF intake and total energy intake was not found. This may partly reflect the association between toddlers’ UPF intake and consumption of CMF, which Gemini parents have previously reported giving toddlers due to concerns over poor appetite [18]. Further research, particularly longitudinal analysis is required to understand associations between UPF intake and adiposity [9].

In this paper UPF was used to characterise children’s diets. However, no evaluation was made of the effects of consuming any single food or UPF sub-group and it is recognised that these vary considerably in nutrient profile [32]. In line with previous research including children and adults in countries with varied income levels, higher UPF consumption at both time points was associated with increased intake of free sugar and sodium [24, 29, 33]. Free sugar intake among toddlers in the highest quintile for UPF was double that of those in the lowest quintile; in mid-childhood individuals in the highest UPF quintile consumed approximately 1.5 times more free sugar than those in the lowest quintile. Toddlers in all quintiles of UPF intake exceeded the UK free sugar maximum recommendation of 5%E, reflecting the high intake of UPF even among those in the lowest quintile (27.9%E) [1]. Toddlers in the two highest UPF quintiles also exceeded the more lenient UK population maximum recommendation of 10%E from free sugar [26]. Similarly in mid-childhood, the lowest UPF consumers received 40.9%E from UPF and individuals in all quintiles exceeded the maximum of 10%E from free sugar [26]. High free sugar intake increases the risks of dental caries and contributes to excess energy intake and is particularly concerning in toddlerhood when lifelong eating habits are becoming established [1, 6]. The negative association between UPF and protein intake in toddlerhood is consistent with findings in other populations but the diets of children in all UPF quintiles satisfied protein requirements [11, 27].

Many of the sugary UPFs consumed are easily recognisable as discretionary or noncore foods (puddings, ice cream, cookies and confectionery) which collectively contributed 4.1% energy to toddlers in Q1 and 12.6% to those Q5, and 11.0% and 23.2% in Q1 and Q5 respectively in mid-childhood (Table 2). Other UPFs, such as children’s yogurts and breakfast cereals, are often marketed as healthy, and while they may provide micronutrients and fibre, they are often a source of free sugar [1]. CMF is a particular concern as it contributes 50% of free sugar intake among UK consumers aged 1-1.5 years [1]. There is no evidence CMF offers any benefits over cows’ milk, which is much cheaper and is the recommended milk for toddlers from 12 months if they are not breastfed [18, 34].

Toddlers in Q5 consumed a third more sodium (32%) than their counterparts in Q1, while children in Q5 consumed a quarter more sodium (24%) than those in Q1. The positive associations between UPF and sodium intakes at both time points reflect observed associations between UPF intake and intake of savory snacks and processed meat. However, individuals in every UPF quintile exceeded the age-appropriate sodium Reference Nutrient Intake (RNI), reflecting high salt levels in UPFs which are UK staples such as bread and breakfast cereal as well as foods such as cheese [28]. Experimental studies with infants and young children suggest they learn to like salt, particularly in specific foods where it is usually found and once this liking is established, it is challenging to alter in later life [6].

In direct contrast to observations in populations including adults and children together, higher UPF consumption in toddlerhood was associated with lower intake of fat and saturated fat [11]. This negative association is largely explained by high intakes of cows’ milk among toddlers consuming less UPF. As population guidelines for fat and saturated fat don’t apply in full until children reach 5 years of age any health implications are unclear. No association was seen between UPF and fat or saturated fat in mid-childhood reflecting the UPF sub-groups commonly marketed for children, which are predominantly sweet foods, such as cookies, desserts and confectionery, rather than higher fat UPFs such as fast food and savoury snacks.

In mid-childhood, mean fibre intake was below the RNI [26]. A negative association was found between UPF and fiber intakes in mid-childhood, in line with observations reported in other child and adult populations [11, 12]. However, in toddlerhood, a negative association was only observed when toddlers consuming CMF were excluded (supplementary material 2, Table S2). This and the closer association between UPF intake in toddlerhood and mid-childhood among non-CMF consumers, compared to CMF consumers, suggests that CMF inclusion should be considered carefully when characterising toddlers’ dietary patterns using UPF, depending on the research question. Likewise, higher intakes of cows’ milk among young children with low intakes of other unprocessed or minimally processed foods, such as fruit and vegetables is also pertinent and may explain the lack of an association between UPF and fibre intakes shown here and previously reported for UK pre-school children [12].

The association between UPF intakes in toddlerhood and mid-childhood aligns with previous evidence suggesting dietary trajectories are set early in life. For example, a dietary pattern characterized by ‘processed and fast foods’ was shown to track moderately between the ages of 2 and 5 years in the French EDEN cohort (r = 0.35, p < 0.001, n = 989) [35]. Our analyses provide a comprehensive insight into UPF intake trends from toddlerhood to mid-childhood. As children matured a rise in dietary share in UPF was seen. Furthermore, an increase in dietary share of UPF was accompanied by a reduction in intake of unprocessed and minimally processed foods in favour of ready-to-eat and ready-to-heat products, including pizza, processed meat, savoury snacks, puddings and sweet cereal products, desserts, and confectionery. It has been suggested that the hyperpalatable nature of some UPF may partly drive continued consumption of these foods, which goes beyond habit formation [32, 36]. A Brazilian study found UPF intake at 4 years of age was associated with higher food responsiveness (eating in response to external food cues) at 7 years, however, intake and appetitive traits may be bidirectional and therefore this requires further investigation [14].

UPFs classified as infant foods and drinks provided less than 2%E for toddlers, compared to UK national survey data showing commercial infant foods and drinks provided 6%E among younger toddlers (12–18 months) [1]. When applying Nova classification to foods consumed by toddlers in the current study, a range of commercial products were identified that didn’t meet criteria for UPF classification but mimicked UPFs, including infant ready meals and infant snacks resembling cookies and flavoured maize (corn) puffs. Early exposure to these processed foods is unlikely to encourage consumption of vegetables, which are generally less popular although liking may be increased by repeated early exposure [1, 37, 38].

Foods marketed for children in the UK, for example breakfast cereals with cartoon characters, are often less healthy options, with many meeting the criteria for classification as high fat, salt or sugar (HFSS) [39]. Across Europe, UPFs marketed for children typically have a worse nutrient profile – i.e. more fat, saturated fat, sugar and sodium – compared with less processed products [17]. Restricting promotion of HFSS products is currently proposed in the UK to address childhood obesity, partly by prompting reformulation [40, 41]. However, foods that fall just below the threshold for HFSS but comprise a large proportion of children’s diets, such as some breakfast cereals, are a concern. Integrated, far reaching policies would be needed to redress the balance of children’s diets toward a lower proportion of UPF, such as adding warning labels to products, inclusive school food policies and subsidies on fresh and minimally processed food [29, 42, 43]. While an increasing number of countries recognize UPF in national guidelines and are taking a policy approach to limit intake, the UK’s Scientific Advisory Committee on Nutrition (SACN) are adopting a more cautious approach, as indicated in a recent statement, and plan to reconsider existing and new evidence in 2024 [44].

The results of the present study should be considered in the light of methodological strengths and limitations. Firstly, in Gemini, as in many cohort studies, there is an over-representation of parents of white ethnicity with a higher SES, particularly in mid-childhood, compared with the UK population. Parent reports of children’s diets come with inherent limitations, for example, foods not consumed in the presence of the diary keeper, such as in school, may be less accurately reported. The lower energy intake recorded when children were 7-years-of-age, compared to the EAR (7 to10-years-of-age), may reflect underreporting, which is common in dietary surveys, as well as age, as children were at the lower end of the EAR age range [25]. Furthermore, data were collected in 2009/10 and 2014/15 so may underestimate current UPF intakes nationally. The Nova classification system has been criticized for grouping together foods with differing nutritional attributes and because some research groups report lack of inter-assessor agreement, which was minimized in the current study by utilizing multiple coders [45, 46]. The dietary assessment software used (DINO) was not specifically designed for Nova classification and a pragmatic approach was necessary, which may have resulted in systematic errors. Some simple composite items, such as fried mushrooms, were coded in DINO as a single item and the decision was made to classify these as Nova 1, resulting in the small amount of cooking oil not being accounted for in the estimation of culinary ingredients (Nova 2) and energy from Nova 1 being overestimated. Another issue arose with composite dishes such as lasagne, which were generally reported with brand information (if commercially produced) or recipe details (if homemade), but a small number of entries lacked these details, so these were classified as Nova 4. The results presented in Tables 2 and 3 don’t represent a direct comparison between diets at each age as only 592 diet records were completed in mid-childhood compared with 2,591 in toddlerhood. As there is a dearth of data published for very young children, this was considered more appropriate than disregarding data for 1,999 toddlers. Analyses of dietary change (Tables 4 and 5) included only the 570 children with diet records at both ages.

In conclusion, UPF intake was high in toddlerhood and mid-childhood in the UK and recommended free sugar and sodium limits were exceeded. In toddlerhood the principal UPFs consumed were those typically marketed as healthy options, including flavoured yogurts and higher-fiber breakfast cereals, whereas in mid-childhood puddings and sweet cereal products and white breads were most common. Higher UPF intake at both time points was associated with higher free sugar and sodium intakes, and in mid-childhood, a negative association with fiber was observed. UPF intake in toddlerhood was predictive of UPF intake in mid-childhood. Integrated policies are needed to provide children with diets with fewer ultra-processed foods in order to lay the foundations for a healthy diet in adulthood.

## Electronic supplementary material

Below is the link to the electronic supplementary material.

Supplementary Material 1

Supplementary Material 2

## Data Availability

Anonymized data in the manuscript, code book and analytic code can be made available upon reasonable request after publication. Requests can be made by downloading and completing the data access form available through the Gemini study website which further details the data sharing process (https://www.geministudy.co.uk/data-access). Requests will be reviewed by the Gemini Executive Committee in accordance with the Gemini Data Access Policy.
