# Supplementary material for: Ultra-processed food intake in toddlerhood and mid-childhood in the UK: cross sectional and longitudinal perspectives
Source: Eur J Nutr. 2024 Oct 4;63(8):3149–60. doi: 10.1007/s00394-024-03496-7 (PMC11519182; doi:10.1007/s00394-024-03496-7)
Supplement: Supplementary file 1 — Supplementary Material 1 [file 394_2024_3496_MOESM1_ESM.docx]

**Ultra-processed food intake in toddlerhood and mid-childhood in the UK: cross sectional and longitudinal perspectives**

Rana E. Conway^1^, Gabriella N. Heuchan^1^, Lisa Heggie^1^, Fernanda Rauber^2,3^, Natalie Lowry^1^, Hannah Hallen^1^, Clare H. Llewellyn^1^

^1^Research Department of Behavioral Science and Health, University College London, London, UK. ^2^Centre for Epidemiological Research in Nutrition and Health, University of São Paulo, São Paulo , Brazil

^3^Department of Preventive Medicine, School of Medicine, University of Sao Paulo, Sao Paulo, Brazil.

**Corresponding author**: Dr Rana Conway, [r.conway@ucl.ac.uk](mailto:r.conway@ucl.ac.uk)

Classifying foods and drinks consumed by twins in the Gemini cohort (age 21 months and age 7 years) using the Nova system

Foods were classified using Nova (not an acronym) system based on the list of ingredients and product name, as described by Monterio^123^

| NOVA | Item | Notes |
| --- | --- | --- |
| 1 | cocoa powder, decaffeinated tea & coffee, lactose free milk, gluten free flour | Similar to tea, coffee, no additional ingredients (FR advice) |
|  | Baby foods inc. porridge, muesli, purees in jars, pouches or boxes made only from items in group 1 | (FR advice) |
|  | Homemade dishes made only, or mainly, with items in group 1  e.g. scrambled egg, porridge made with all whole milk, fruit cooked with sugar, pancakes, Yorkshire pudding, mashed potato, soup, shepherd’s pie | should ideally be coded as separate items, only coded this way if insufficient detail provided in diary |
|  | Meat or vegetables cooked in fat as e.g. fried chicken without coating, fried tomato (in Gemini fat is generally coded separately so weight specified is for the meat or vegetable without fat) | majority of weight is from the vegetable or meat |
|  | Fruit & veg tinned in juice, without salt or sugar | Ingredients are all group 1 (FR advice) |
|  | Fruit juice (fresh, canned or without additional detail) – expect cranberry or passion fruit juice | Ingredients are all group 1 (FR advice |
|  | Egg pasta and noodles, retail or no other details | Just egg & wheat flour |
|  | Foods with just one ingredient but industrially produced, e.g. puffed wheat, shredded wheat, corn thins/cakes, Matzo crackers, pasta | Classified according to ingredients list |
|  | Freeze dried fruit or powdered vegetables | Only ingredient is fruit or veg so doesn’t make breakfast cereals or fruit bars automatically Nova 4 (FR advice) |
| 2 | Baking powder |  |
| 3 | Baby food jars or pouches of NOVA 1 & NOVA 2 (e.g. baby meal of beef, veg & oil) | Commercial preparation with Nova 1 and Nova 2 ingredients |
|  | Baby savoury puffs/crisps made with onion/tomato powder, potato starch (without obvious UPF ingredients) | Commercial preparation with Nova 1 and Nova 2 ingredients |
|  | Retail products made mainly with group 1 items but including some group 2 e.g. vegetables prepared for roasting, with oil | Commercial preparation with Nova 1 and Nova 2 ingredients |
|  | Homemade dishes where approx. half or more than half of weight on ingredients are NOVA 2 items, e.g. sponge cake made with butter, homemade apple sauce, homemade chutney | Commercial preparation with Nova 1 and Nova 2 ingredients |
|  | Homemade dishes with pastry e.g. pies, sausage rolls | Likely to be made with commercially prepared pastry |
|  | Soup, described as fresh or sold in a carton | Commercial preparation with Nova 1 and Nova 2 ingredients |
| 4 | Complete meals e.g. lasagne, curry, stew unless specified homemade | could be homemade or retail & in GEMINI parents tended to list ingredients if homemade |
|  | Pies, flans and quiches, not described as homemade | could be homemade or retail & in GEMINI parents tended to list ingredients if homemade |
|  | Soup, unless specified homemade or fresh |  |
|  | Bread, cakes, pancakes and biscuits, unless specified homemade or other indication dish is homemade e.g. ‘pancakes made with whole milk’ |  |
|  | Anything in breadcrumbs e.g. chicken, cauliflower |  |
|  | Sauces e.g. white sauce, cheese sauce, onion sauce (except homemade apple sauce) | could be jar or packet made up with milk |
|  | Gravy, unless specified ‘homemade’ or ‘thickened’ (meaning made with meat juices & corn flour) |  |
|  | passion fruit juice or cranberry juice | contain non-sugar sweetener and other ingredients |
|  | Baby snacks containing concentrated fruit juice, except jars or pouches with a small amount of conc lemon juice | Conc. Fruit juice is a marker of UPF |
| Not classified | Dietary supplements, such as vitamins, minerals and fish oils | These were excluded from this analysis. |
| All Nova groups | Fortification with vitamins and/or minerals did not alter NOVA classification | FR advice |

FR = Fernanda Rauber

1. Monteiro C, Cannon G, Levy R. Ultra-processed foods: What they are and how to identify them. *Public Heal Nutr*. 2019;22(5):936-941. doi:10.1017/s1368980018003762

2. Monteiro CA, Cannon G, Levy R, et al. NOVA. The star shines bright. *World Nutr*. 2016;7(1-3):28-38.

3. Monteiro CA, Cannon G, Moubarac JC, Levy RB, Louzada MLC, Jaime PC. The UN Decade of Nutrition, the NOVA food classification and the trouble with ultra-processing. *Public Health Nutr*. 2018;21(1):5-17. doi:10.1017/S1368980017000234
