# Supplementary material for: Ultra-processed food intake in toddlerhood and mid-childhood in the UK: cross sectional and longitudinal perspectives
Source: Eur J Nutr. 2024 Oct 4;63(8):3149–60. doi: 10.1007/s00394-024-03496-7 (PMC11519182; doi:10.1007/s00394-024-03496-7)
Supplement: Supplementary file 2 — Supplementary Material 2 [file 394_2024_3496_MOESM2_ESM.docx]

**Ultra-processed food intake in toddlerhood and mid-childhood in the UK: cross sectional and longitudinal perspectives**

Rana E. Conway^1^, Gabriella N. Heuchan^1^, Lisa Heggie^1^, Fernanda Rauber^2,3^, Natalie Lowry^1^, Hannah Hallen^1^, Clare H. Llewellyn^1^

^1^Research Department of Behavioral Science and Health, University College London, London, UK. ^2^Centre for Epidemiological Research in Nutrition and Health, University of São Paulo, São Paulo , Brazil

^3^Department of Preventive Medicine, School of Medicine, University of Sao Paulo, Sao Paulo, Brazil.

**Corresponding author**: Dr Rana Conway, [r.conway@ucl.ac.uk](mailto:r.conway@ucl.ac.uk)

**Supplementary Table S1. Contribution of UPF sub-groups to total energy (mean ± SD) for total sample and by quintile of UPF intake in toddlerhood (21 months) excluding participants with >0 kcals from Commercial Milk Formula milk (n=2136)**

| UPF sub-groups | Q1  (n=427) | Q2  (n=427) | Q3  (n=428) | Q4  (n=428) | Q5  (n=426) |
| --- | --- | --- | --- | --- | --- |
| Higher fiber breakfast cereals | 3.4 ± 3.0 | 4.6 ± 3.9 | 5.0 ± 4.1 | 4.3 ± 3.7 | 4.4 ± 4.3 |
| Lower fiber breakfast cereals^b^ | 0.6 ± 1.4 | 0.7 ± 1.4 | 0.9 ± 1.7 | 1.3 ± 2.4 | 1.2 ± 2.1 |
| Wholegrain breads | 3.6 ± 3.6 | 4.3 ± 3.9 | 4.3 ± 4.2 | 4.0 ± 4.5 | 3.9 ± 5.3 |
| White breads^b^ | 2.1 ± 2.8 | 2.8 ± 3.6 | 3.6 ± 4.3 | 4.7 ± 4.6 | 6.0 ± 5.6 |
| Flavoured yogurt^b^ | 2.9 ± 3.0 | 4.3 ± 3.2 | 4.4 ± 3.4 | 5.1 ± 3.6 | 5.1 ± 4.0 |
| Infant foods and beverages | 1.8 ± 3.1 | 1.9 ± 3.0 | 1.7 ± 3.1 | 1.6 ± 3.5 | 1.5 ± 4.4 |
| Puddings and sweet cereal products^b^ | 1.4 ± 2.6 | 2.4 ± 3.4 | 3.3 ± 4.1 | 4.1 ± 4.6 | 4.2 ± 5.2 |
| Ice cream, dairy desserts and ice lollies^b^ | 0.5 ± 1.3 | 0.7 ± 1.5 | 1.1 ± 2.0 | 1.3 ± 2.3 | 1.9 ± 3.2 |
| Biscuits^b^ | 1.5 ± 2.4 | 2.1 ± 2.5 | 2.6 ± 3.0 | 3.3 ± 3.3 | 5.3 ± 5.4 |
| Confectionary^b^ | 0.5 ± 1.2 | 0.8 ± 2.1 | 1.3 ± 2.3 | 2.1 ± 2.9 | 2.7 ± 3.7 |
| Sweet spreads^b^ | 0.2 ± 0.6 | 0.4 ± 0.8 | 0.5 ± 1.3 | 0.6 ± 1.2 | 0.8 ± 1.8 |
| Savoury snacks^b^ | 1.1 ± 1.8 | 1.3 ± 1.9 | 2.1 ± 2.6 | 2.7 ± 3.0 | 3.9 ± 3.8 |
| Processed meat^b^ | 0.9 ± 1.7 | 1.1 ± 1.9 | 1.7 ± 2.5 | 1.9 ± 2.6 | 2.8 ± 3.9 |
| White meat products & dishes^b^ | 0.1 ± 0.8 | 0.2 ± 0.9 | 0.5 ± 1.5 | 0.8 ± 1.8 | 1.6 ± 3.0 |
| Fish & fish dishes^b^ | 0.7 ± 1.6 | 1.2 ± 2.0 | 1.4 ± 2.2 | 1.4 ± 2.1 | 2.1 ± 2.3 |
| Potato products, e.g. chips, wedges, instant mash^b^ | 0.4 ± 1.4 | 0.7 ± 1.4 | 0.9 ± 2.0 | 1.4 ± 2.3 | 2.6 ± 3.4 |
| Pizza^b^ | 0.2 ± 1.2 | 0.5 ± 1.8 | 0.4 ± 1.7 | 0.8 ± 2.7 | 1.3 ± 3.6 |
| Savoury pastry dishes^b^ | 0.2 ± 0.9 | 0.6 ± 1.9 | 0.6 ± 1.7 | 0.8 ± 2.1 | 1.5 ± 4.0 |
| Spreadable fats^b^ | 0.8 ± 1.3 | 1.5 ± 1.9 | 2.1 ± 2.2 | 2.6 ± 2.7 | 3.1 ± 3.1 |

SD, standard deviation; UPF, ultra-processed food

^a^UPF sub-groups contributing >1% energy included, therefore sum of sub-groups does not yield the value of UPF contribution.

^b^Positive linear association between % of total energy from UPF (continuous) and % of total energy from UPF sub-group (continuous), P<0.001.

**Supplementary Table S2**.  **Daily energy and nutrient intake (mean ± SD) according to quintile of UPF intake in toddlerhood (21 months) excluding participants with >0 kcals from Commercial Milk Formula (n=2136)**

|  | Quintile of UPF intake | | | | |
| --- | --- | --- | --- | --- | --- |
|  | Q 1  (n=427) | Q 2  (n=427) | Q 3  (n=428) | Q 4  (n=428) | Q 5  (n=426) |
| UPF (%E) | 26.8 ± 5.4 | 36.9 ± 1.8 | 43.6 ± 2.01 | 51.0 ±2.4 | 65.2 ± 8.0 |
| Energy (kcals) | 1024 ± 178 | 1040 ± 173 | 1035 ± 169 | 1053.61 ± 206.41 | 1010 ± 212 |
| Fat (%E)^b^ | 37.6 ± 5.6 | 36.6 ± 4.6 | 36.6 ± 4.3 | 36.2 ± 4.7 | 35.0 ± 4.9 |
| Saturated fat (%E)^b^ | 17.4 ± 3.6 | 16.5 ± 3.3 | 16.3 ± 3.0 | 15.9 ± 3.1 | 14.3 ± 3.4 |
| Protein (%E)^b^ | 16.6 ± 2.3 | 16.5 ± 1.7 | 16.2 ± 1.9 | 15.3 ± 1.8 | 14.4 ± 2.0 |
| Carbohydrate (%E)^a^ | 45.9 ± 5.8 | 47.0 ± 4.9 | 47.3 ± 4.5 | 48.5 ± 5.0 | 50.7 ± 5.4 |
| Free sugar (%E)^a^ | 6.1 ± 3.5 | 7.5 ± 3.4 | 8.1 ± 3.1 | 9.6 ± 3.8 | 10.9 ± 4.2 |
| Fiber (g)^b^ | 9.5 ± 2.9 | 9.6 ± 3.0 | 9.1 ± 2.8 | 8.7 ± 2.7 | 8.6 ± 2.8 |
| Sodium (mg)^a^ | 918 ± 261 | 1047 ± 239 | 1156 ± 284 | 1212 ± 334 | 1332 ± 377 |

SD, standard deviation; %E, percentage of total energy; Q, quintile; UPF, ultra-processed food

^a^ Positive linear association between % energy from UPF (continuous) and nutrient (continuous), P<0.001

^b^ Negative linear association between % energy from UPF (continuous) and nutrient (continuous), P<0.001
